# Diagnostic Performance of Contrast-Enhanced Ultrasound in the Evaluation of Small Renal Masses: A Systematic Review and Meta-Analysis

**DOI:** 10.3390/diagnostics12102310

**Published:** 2022-09-25

**Authors:** Antonio Tufano, Luca Antonelli, Giovanni Battista Di Pierro, Rocco Simone Flammia, Rocco Minelli, Umberto Anceschi, Costantino Leonardo, Giorgio Franco, Francesco Maria Drudi, Vito Cantisani

**Affiliations:** 1 Department of Maternal-Infant and Urological Sciences, “Sapienza” Rome University, Policlinico Umberto I Hospital, 00185 Rome, Italy; 2 Department Life and Health “V. Tiberio”, University of Molise, Francesco De Sanctis 1, 86100 Campobasso, Italy; 3 Department of Urology, IRCCS “Regina Elena” National Cancer Institute, Via Elio Chianesi 53, 00144 Rome, Italy; 4 Department of Radiological, Oncological and Pathobiological Sciences, Sapienza University of Rome, 00144 Rome, Italy

**Keywords:** contrast-enhanced ultrasound (CEUS), renal cancer, renal masses, small renal masses, small kidney masses, perfusion, quantitative analysis

## Abstract

Ultrasound (US) is a highly sensitive imaging tool in the detection of renal masses. However, the detection rate of small renal masses (SRMs) (<4 cm) is still limited. In this scenario, contrast-enhanced ultrasound (CEUS) is a relatively novel, but increasingly utilized, diagnostic modality which aims to increase the overall diagnostic ability in the identification of SRMs. In consequence, we performed a systematic review (SR) and pooled meta-analysis to investigate the diagnostic performance of CEUS in the evaluation of SRMs confirmed by pathology. A SR up to April 2022 was performed according to the Preferred Reporting Items for Systematic Reviews and Meta-Analyses (PRISMA) statement. The diagnostic performance of CEUS was evaluated basing on malignant vs. benign SMRs. Sensitivity, specificity, positive predictive value (PPV), and negative predictive value (NPV) from eligible studies were pooled, and summary receiver operating characteristic (SROC) curves were constructed for each endpoint. Overall, five qualified studies were deemed suitable for this meta-analysis. Finally, diagnostic performance of CEUS showed an accuracy of 0.93 in detecting malignant masses (sensitivity of 0.94, PPV of 0.95, specificity of 0.78, and NPV of 0.73). Taken together, CEUS may represent a promising minimally invasive diagnostic tool for characterization of SMRs, since it allows clinicians to identify malignant lesions.

## 1. Introduction

The progressive use of modern imaging procedures has led to an increased detection rate of small renal masses (SRMs) (≤4 cm) [1]. Interestingly, there is an inverse correlation between kidney lesion diameter and malignancy [2]. More specifically, benign lesions represent up to 25% of SRMs after final histopathological examination [3]. To date, preoperative renal imaging tools are commonly based on contrast-enhanced CT (CECT) or contrast-enhanced MRI. However, limitations of these techniques, such as radiation exposure, renal toxicity, and allergies to contrast agents, should be considered [4]. In this scenario, with the recent advances in ultrasound (US) technology, contrast-enhanced US (CEUS) has emerged as a fast, reliable, and cost-effective imaging tool in the preoperative assessment of renal masses. The use of microbubble contrast agents allows the study of micro- and macro-vascularization and dynamic enhancing patterns for better characterization of indeterminate lesions [5]. Further advantages of CEUS are represented by the lack of renal excretion of contrast agents and their use in patients presenting renal insufficiency [6]. Moreover, several studies revealed a non-inferiority of CEUS with regard to CECT and MRI in the diagnosis of renal masses [7,8]. As demonstration of this concept, EFSUMB guidelines recommend the use of CEUS to characterize kidney lesions (LoE 1b) [9].

Basing on this hypothesis, we aimed to systematically review the current literature and meta-analyze the diagnostic accuracy of CEUS in differentiation between benign and malignant SRMs proved pathologically.

## 2. Materials and Methods

### 2.1. Evidence Acquisition

This study was performed according to the 2020 Preferred Reported Items for Systematic Reviews and Meta-analysis (PRISMA) guidelines [10]. All available studies were retrospective observational cohorts, and for this reason we followed the guidelines for meta-analysis of observational studies (MOOSE).

### 2.2. Literature Search

A comprehensive systematic review of the literature was performed up to April 2022 by searching PubMed, Embase, Cochrane Central Search Library, and Web of Science with no restriction of time. We relied on the population, intervention, comparison, outcome, and study design principle (PICOS) to define study eligibility.

- Population—patients with US diagnosis of SMRs
- Intervention—CEUS
- Comparator— final histopathological examination
- Outcome—diagnostic accuracy of CEUS (sensitivity, specificity, accuracy, positive predictive value, and negative predictive value)
- Study design—prospective and retrospective cohort studies

We excluded reviews, case reports, study protocols, editorials, abstracts, replies from the authors, and articles not published in English language. Search strategy relied on the combination of the following terms: “small renal masses”, “small kidney lesions”, “renal mass”, “renal cancer” “renal tumor”, “renal carcinoma”, “kidney mass”, “kidney cancer”, “kidney tumor”, “kidney carcinoma”, “CEUS”, contrast-enhanced ultrasound”, “diagnosis”, diagnostic”, “sensitivity”, “specificity” and “accuracy”. Search results were independently reviewed by two authors (A.T., L.A.). Possible conflicts were resolved by discussion or with an independent arbiter (V.C.). Full-text articles were retrieved for further qualitative and quantitative review.

### 2.3. Risk of Bias Assessment

Two independent reviewers assessed the methodological quality, including risk of bias and applicability of each study, according to the Quality Assessment of Diagnostic Accuracy Studies-2 (QUADAS-2). Assessment of study bias and applicability was evaluated as low, high, or unclear (Table 1). Disagreements were resolved through a discussion and, if necessary, arbitration by another reviewer.

### 2.4. Data Collection

We constructed 2 × 2 contingency tables for each of the included studies and calculated sensitivity, specificity, positive predictive value (PPV), negative predictive value (NPV), diagnostic accuracy, and corresponding 95% confidence intervals (CIs) of CEUS in diagnosing SRMs as benign or malignant. Other descriptive variables extracted were: study design, time, number of patients, mean age, tumor size, histology, dose of contrast injected, and number/experience of readers.

### 2.5. Statistical Analysis

To address diagnostic performance of CEUS, sensitivity, specificity, PPV, and NPV from different studies were pooled and summary receiver operating characteristic (SROC) curves were constructed.

We relied on a bivariate random-effects model for analysis and pooling of the diagnostic performance measures across studies. The bivariate model estimates pairs of logit-transformed sensitivity and specificity from studies, incorporating the correlation that might exist between sensitivity and specificity [11].

The heterogeneity was assessed using the Q test and I^2^ statistic. I^2^ ≤ 25% indicated low heterogeneity, 25% < I^2^ ≤ 50% indicated mild heterogeneity, 50% < I^2^ ≤ 75% indicated moderate heterogeneity and I^2^ > 75% indicated high heterogeneity [12].

All analyses were performed using the R statistical package v3.6.1.

Statistical analysis was two-sided and statistical significance was set at *p* < 0.05.

## 3. Results

### 3.1. Characteristics of Included Studies

**Figure 1:**
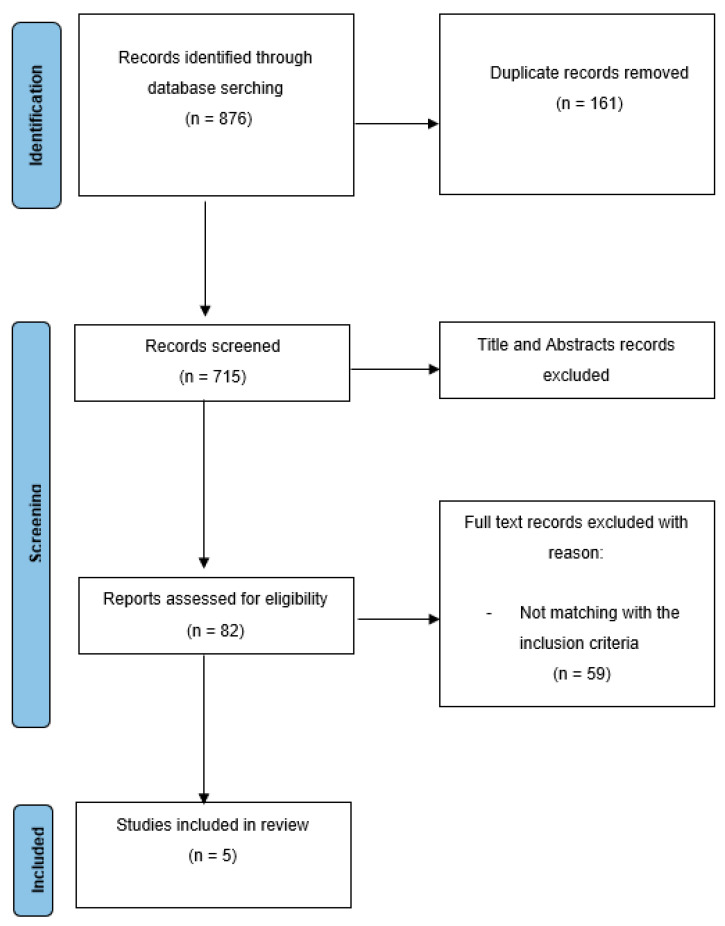
Study flow chart.

The initial literature search yielded a total of 876 studies. Among them, 161 were removed due to duplication, and 715 articles were included for title and abstract screening. Subsequently, 82 records were identified for further full-text evaluation. Finally, five qualified studies were deemed suitable for this meta-analysis (Figure 1).

Table 2 summarizes the characteristics of the eligible studies.

All studies were retrospective and published between 2014 and 2022. Overall, 514 patients for a total of 517 SMRs were analyzed in this meta-analysis. The mean patient age ranged from 52.6 to 61 years. Mean tumor size ranged from 1.8 to 2.9 cm and from 1.7 and 2.8 for malignant and benign tumors, respectively. The most common malignant and benign histopathological specimens were represented by clear cell RCCs and angiomyolipomas, respectively. A total of four studies reported doses of contrast agent used, ranging from 1.6 mL to 2.4 mL. Moreover, three studies relied on two radiologist readers, while two had a single reader. Finally, inter-observer agreement ranged from 0.74 to 0.89, when reported.

### 3.2. Accurancy of CEUS in Distinguishing Malignant from Benign Small Renal Masses

**Figure 2:**
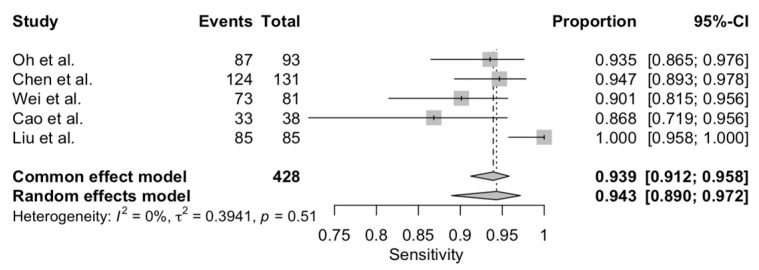
Pooled sensitivity of CEUS in detecting small renal masses.

**Figure 3:**
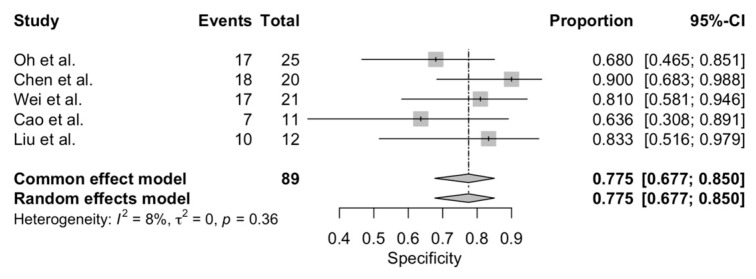
Pooled specificity of CEUS in detecting small renal masses.

**Figure 4:**
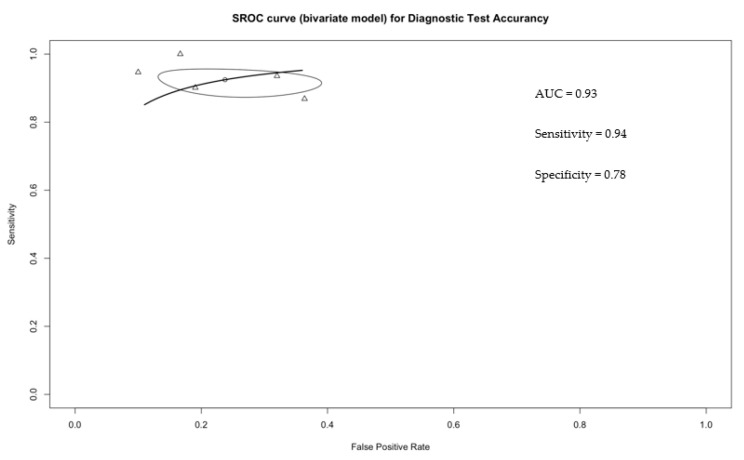
SROC curve (bivariate model) of CEUS in detecting small renal masses.

The pooled sensitivity and specificity were 0.94 (95% CI: 0.89–0.97) and 0.78 (95% CI: 0.68–0.85), respectively (Figure 2 and Figure 3). Additionally, pooled PPV and NPV were 0.95 (95% CI: 0.93–0.97) and 0.73 (95% CI: 0.63–0.81), respectively. Supplementary Table S1. summarizes predicted values calculated from the eligible studies. Finally, the bivariate random effect ROC curve depicted a diagnostic accuracy of 0.93 (Figure 4).

## 4. Discussion

The value of CEUS in the preoperative imaging setting has been described in multiple studies [13,14,15]. In particular, the application of this tool in the evaluation of kidney masses has historically represented a fascinating and intriguing challenge for the urological community. In consequence, our aim was to systematically review the role of CEUS as a reliable and accurate imaging modality in the preoperative evaluation of SMRs.

Our analyses led to several noteworthy observations. First, diagnostic performance of CEUS in detecting malignant vs. benign SMRs showed an accuracy of 0.89 with a sensitivity of 0.94, a specificity of 0.78, a PPV of 0.95, and a NPV of 0.73. In consequence, CEUS may represent a reliable tool to detect potentially malignant SRMs. This result is mainly imputable to the study from Liu et al., which relied on an adequate sample size (*n* = 97) and reported a sensitivity of 1.00 (95% CI: 0.96–1.00) [16].

Second, in the cohort described by Oh et al., diffuse heterogeneous enhancement, late washout, and perilesional rim-like enhancement were the most common findings for RCCs. These characteristics depicted an accuracy of 0.82 in detecting RCCs [17]. Specifically, the presence of perilesional rim-like enhancement, commonly defined as pseudocapsule, results from tumor growth producing compression, ischemia, and necrosis of adjacent normal tissue, and consequent change in fibrous tissue. Notably, this sign was useful in discriminating malignancy among other historical series [18,19]. However, it should be taken into account that the presence of pseudocapsule could be inversely related to the tumor size of RCCs and is usually absent in hemorrhagic cysts and angiomyolipomas [20,21,22,23,24].

Third, when focusing on heterogeneous enhancement, the rapid growth of the tumor, RCC is prone to ischemia, necrosis, and cystic change, which might explain the heterogeneous enhancement on CEUS [25]. However, in the cohort of Cao et al., only 32.8% RCCs showed heterogeneous enhancement, with no difference among the three RCC subtypes evaluated (clear cell, papillary, and chromophobe) [26]. A possible explanation might be that the included population relied only on patients with a tumor diameter of 4 cm or less. Conversely, Oh et al. found that 78.9% of their cohort exhibited heterogeneous enhancement [17]. The above observations highlight how this feature is not reliable and differs with regard to tumor size. Interestingly, Liu et al. evaluated the usefulness of CEUS features in the evaluation of small clear cell renal cell carcinoma (sccRCC). Although the authors only relied on the incidence of CEUS characteristics, without discerning among potentially benign or malignant renal masses, the most common pattern for sccRCC was hyper-iso-enhancement (94.1%) and fast wash-in (87.5%) [27].

Fourth, on contrast-enhanced exams, oncocytoma may appear with a central stellate scar or with a spoke-wheel pattern of vascularity [28]. However, sensitivity or specificity reported is still scarce. Interestingly, Wei et al. was the only study including oncocytomas [29]. Here, all three lesions were misdiagnosed by CEUS as RCCs. Although based on a small sample size, imaging diagnosis of small oncocytomas is confirmed to be challenging, making oncocytoma one of the most commonly excised benign renal lesion [30].

Fifth, when focusing on AMLs, specificity ranged from 0.64 to 0.91. AMLs often show iso-enhancement characteristics at peak time on qualitative CEUS, which makes them difficult to be distinguished from clear cell RCCs [21]. Moreover, imaging features may overlap between hypo-vascular benign and malignant renal lesions, such as papillary RCCs and lipid poor AMLs. Interestingly, in the study by Wei et al., a. total of 20 AMLs (including 10 classic AMLs and 10 lipid poor AMLs) were included [29]. Here, 16 cases were correctly diagnosed by CEUS and 4 (all lipid poor AMLs) were misdiagnosed as malignant lesions (RCCs). However, due to heterogeneity reported in previous series and the limited sample size of patients with AMLs analyzed in the current study, further larger cohorts are necessary to evaluate the imaging characteristics of AMLs in CEUS and potentially detect reliable features which may allow for safe discrimination from RCCs.

Taken together, CEUS may represent a promising minimally invasive diagnostic tool for characterization of SMRs, since it allows clinicians to identify malignant lesions, showing excellent diagnostic performance rates.

Nevertheless, our study presents several potential limitations. First, CEUS results may be interpreted according to several parameters (hyper- or hypo- enhancement, wash-out, wash-in, peak intensity, etc.); here, we selected only studies whose authors aimed to report CEUS’s accuracy results in the easiest fashion (i.e., only malignant vs. benign). Second, the number of included articles is low. Third, all the eligible studies were retrospective, and some were limited in sample size. Finally, CEUS is an operator-dependent performance skill, with difficulty in reproducibility and sometimes requiring repeated injections. In this systematic review, only three studies relied on more than one reader to interpret images.

## 5. Conclusions

Currently, available evidence suggests potential benefits of CEUS, particularly in the diagnosis of benign and malignant SRMs. As a consequence, routine implementation of CEUS in clinical decision making could, therefore, be considered. However, the number of articles included and of patients investigated are limited, highlighting the need for well-designed, adequately sampled and powered longitudinal studies to reach definitive conclusions.

## Figures and Tables

**Table 1:** Quality assessment of the studies according to QUADAS-2. L low risk of bias, H high risk of bias, U unclear risk of bias.

Risk of Bias					Applicability Concerns		
Study	Patient Selection	Index Test	Reference Standard	Flow and Timing	Patient Selection	Index Test	Reference Standard
Chen et al.	L	L	L	U	L	L	L
Cao et al.	U	L	L	L	U	L	L
Liu et al.	L	L	L	L	L	L	L
Oh et al.	H	L	L	U	H	L	L
Wei et al.	L	L	L	U	L	L	L

**Table 2:** Baseline characteristics of the included studies.

Study	Year	Mono/ Multi	Study Design	Patient, n	Lesion, n	Age, yrs (SD)	Tum Size, cm Mean (SD)	Contrast Agent, mL	Reader, n	Inter-obServer Agreement, K Value (95% CI)	Hystopatological Type
Oh et al.	2014	mono	retrospective	49	49	61	RCCs: 2.89 (±0.81); AMLs: 2.85 (±0.85)	SonoVue	1	N.R.	38 RCCs; 11 AMLs
Chen et al.	2015	mono	retrospective	99	102	RCCs: 56.6 (±16.5); AMLs: 56.6 (±16.5)	RCCs: 1.81 (±0.59); AMLs: 1.77 (±0.52)	SonoVue, 1.2 ml	2	N.R.	81 RCCs (68 clear cell carcinoma, 8 papillary carcinoma, 4 chromophobe carcinoma, 1 collecting duct carcinoma); 21 AML
Wei et al.	2017	mono	retrospective	118	118	Benign: 52.6 (±10.6); Malignant: 52.8 (±11.4)	Benign: 2.76 (±0.76); Malignant: 2.74(±0.73)	SonoVue, 1.6–2.4 mL	2	0.89 (0.79–0.98)	25 benign (25/118, 21.2%): 20 AMLs, 3 oncocytomas, and 2 metanephric adenomas; 93 malignant (93/118,78.8%): 75 clear cell RCCs, 13 papillary RCCs, 2 chromophobe RCCs, 1 unclassified RCC, 1 Xp11.2 translocation/TFE3 gene fusion RCC, and 1 mucinous tubular and spindle cell carcinoma.
Cao et al.	2021	mono	retrospective	151	151	RCCs: 59.8 (±12.0); AMLs: 57.4 (±14.8)	RCCs: 2.7 (±0.8); AMLs: 2.2 (±1.0)	SonoVue, 1.2–2.0 mL	2	0.74	131 RCCs (110 ccRCCs, 12 pRCCs, and 9 chRCCs); 20 AMLs
Liu et al.	2022	mono	retrospective	97	97	RCCs: 60.5 (±12.1); AMLs: 54.8 (±8.6)	RCCs: 2.9 (±0.7); AMLs: 2.3 (±1.0)	SonoVue, 1.6–2.4 mL	1	N.R.	RCCs (71 ccRCCs, 7 pRCCs, 7 chRCCs); 12 AMLs

## Data Availability

All data generated or analyzed during this study are included in this published article.

## Supplementary Materials

The following supporting information can be downloaded at: https://www.mdpi.com/article/10.3390/diagnostics12102310/s1. Table S1: Predictive values extrapolated from eligible studies.
