# Supplementary material for: Diagnostic Performance of Contrast-Enhanced Ultrasound in the Evaluation of Small Renal Masses: A Systematic Review and Meta-Analysis
Source: Diagnostics (Basel). 2022 Sep 25;12(10):2310. doi: 10.3390/diagnostics12102310 (PMC9600471; doi:10.3390/diagnostics12102310)
Supplement: Supplementary file 1 [file diagnostics-12-02310-s001.zip › diagnostics-1939638-supplementary.pdf]

Supplementary Table S1. Predictive values extrapolated from eligible studies.

Study	TP	FP	FN	TN
**Oh et al. [17]**	87	8	6	17
**Chen et al. [24]**	124	3	7	18
**Wei et al. [29]**	73	4	8	17
**Cao et al. [26]**	33	4	5	7
**Liu et al. [27]**	85	2	0	10
